# RASSF8 regulates progression of cutaneous melanoma through nuclear factor-κb

**DOI:** 10.18632/oncotarget.5030

**Published:** 2015-08-17

**Authors:** Jinhua Wang, Wei Hua, Sharon K. Huang, Kun Fan, Ling Takeshima, Ying Mao, Dave S.B. Hoon

**Affiliations:** ^1^ Department of Molecular Oncology, John Wayne Cancer Institute (JWCI), Providence Saint John's Health Center, Santa Monica, CA; ^2^ Department of Neurosurgery, Huashan Hospital, Fudan University, Shanghai, China

**Keywords:** RASSF8, P53, melanoma, methylation, P65

## Abstract

Our group previously demonstrated that the RASSF1 gene has a significant tumor suppressor role in cutaneous melanoma. The RASSF8 gene is a member of the N-terminal RASSF gene family. Previously, we identified RASSF8 (HOJ1, NCBI Gene ID:11228) expression in cutaneous melanoma; however the functional role of RASSF8 in melanoma is not known. RASSF8 expression was assessed in melanoma cell lines and tumors of different AJCC stages. Results indicated that RASSF8 expression was low in metastatic melanoma lines and decreased with melanoma progression. We then explored the mechanism of RASSF8 downregulation in melanoma by assessing methylation of RASSF8 and demonstrated that methylation of RASSF8 gene promoter was higher in advanced than in early stages melanomas. Functional activity of RASSF8 in melanoma lines by knockdown and overexpression of RASSF8 demonstrated that RASSF8 expression significantly inhibited cell growth, cell migration and invasion, whereas knockdown of RASSF8 expression significantly increased cell growth, cell migration and invasion of melanoma cells by increasing expression of P65 and its downstream target IL-6. Moreover RASSF8 was found to induce apoptosis in melanoma cells by activating the P53-P21 pathway, and also *in vivo* studies demonstrated that inhibiting RASSF8 increases the tumorigenic properties of human melanoma xenografts. These results suggest that RASSF8 plays a significant role in suppressing the progression of cutaneous melanoma.

## INTRODUCTION

At the present time, there are ten known members of the Ras-association domain family (RASSF), RASSF1-10, several of which are believed to be tumor suppressor genes. RASSF can be subcategorized into two groups, the classical C-terminal RASSF proteins (RASSF1-6) and the four recently added N-terminal RASSF proteins (RASSF7-10) [1, 2]. RASSF proteins have been implicated in key biological processes, including cell death, cell cycle control, microtubule stability, promoter methylation, vesicle trafficking, and response to hypoxia [2–8].

Some RASSF gene members have been associated with development of melanoma [9]. Our group has previously demonstrated the correlation between transcriptional inactivation of the RASSF1 gene in cutaneous melanoma and hypermethylation of CpG promoter region, and relation to disease progression [10–13]. RASSF2 promoter is reported to be epigenetically down regulated in some malignant melanoma cell lines [8]. RASSF10 promoter hypermethylation is frequent in malignant melanoma but uncommon in nevi [14].

RASSF8 was initially cloned and identified by our group as HOJ1 (NCBI Gene ID:11228) on chromosome12p12.3, and shown to be expressed in several cancers. Later, the gene was further identified as a lung tumor suppressor gene candidate [15]. The biological and physiological roles of RASSF8 in melanoma are poorly understood; consequently there have been no comprehensive reports on RASSF8's expression and its function in melanoma cells up to date.

In the present study, we investigated the expression and functional role of RASSF8 in melanoma. In comparison to early stage melanoma, promoter region methylation in advanced melanoma resulted in lower expression of RASSF8. Both *in vivo* and *in vitro* studies show inhibition of melanoma cells’ growth, migration and invasion as a result of RASSF8 expression downregulating P65. Furthermore, overexpression of RASSF8 lead to G1-S arrest and induced apoptosis of melanoma cell lines by increasing P53 and P21 expression. RASSF8 also inhibited growth of human melanoma xenografts. Altogether, our findings suggest that RASSF8 has a tumor suppressor role in melanoma.

## RESULTS

### RASSF8 expression in melanoma cell lines

**Figure 1:**
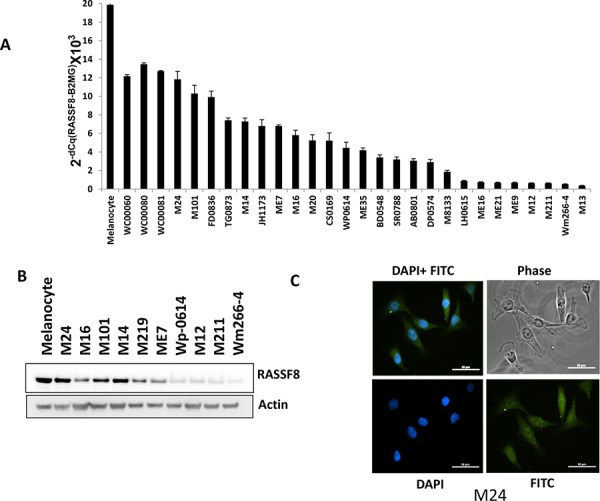
RASSF8 expression in melanoma cell lines **A.** mRNA expression of RASSF8 in melanocyte, 3 primary melanoma cells, 25 melanoma stage III/IV cell lines (Primary melanoma cell lines vs metastasis melanoma cell lines, *p* < 0.05). **B.** Protein expression of RASSF8 in melanocyte and 10 melanoma metastasis cell lines. **C.** RASSF8 was located in the cytoplasm and nucleus of melanoma cells.

To examine RASSF8 mRNA expression variation in cutaneous melanoma cell lines, total RNA was extracted for qRT-PCR from one melanocyte cell line, three primary melanoma cell lines, and 25 metastatic melanoma lines. The results of qRT-PCR analysis were normalized by β2MG (Beta-2-Microglobulin). The results indicated that there was lower RASSF8 expression in metastatic melanoma lines than that in the melanocyte and primary cell lines (Figure 1A). Northern blot analysis using DIG-labeled DNA revealed that RASSF8 mRNA expression was observed in normal tissues, especially ovary and testis tissues (Supplementary Figure 1). The analysis of the Cancer Genome Atlas (TCGA) data also showed significantly lower RASSF8 mRNA expression in systemic melanoma metastasis than in regional lymph node metastasis or primary melanomas (Supplementary Figure 2A). Moreover, western blot analysis confirmed lower RASSF8 protein expression in most of the metastatic melanoma lines (Figure 1B). To assess localization of RASSF8 protein in melanoma cell lines, we performed immunofluorescence (IF) staining. As shown in Figure 1C, RASSF8 protein is present in both the cytoplasm and nucleus of melanoma cells. These results suggest low expression of RASSF8 in most metastatic melanoma cell lines and tissues, decreasing with melanoma progression. To identify specificity of RASSF8 antibody (Ab), we performed IF staining in RASSF8-positive cells (Wm266-4 RASSF8) and RASSF8-negative cells (M24 RASSF8 shRNA). It was shown that RASSF8 is highly expressed in Wm266-4 RASSF8 (Supplementary Figure 3A) and weakly expressed in M24 RASSF8 shRNA (Supplementary Figure 3B).

### Functional activity of RASSF8 in melanoma cells

**Figure 2:**
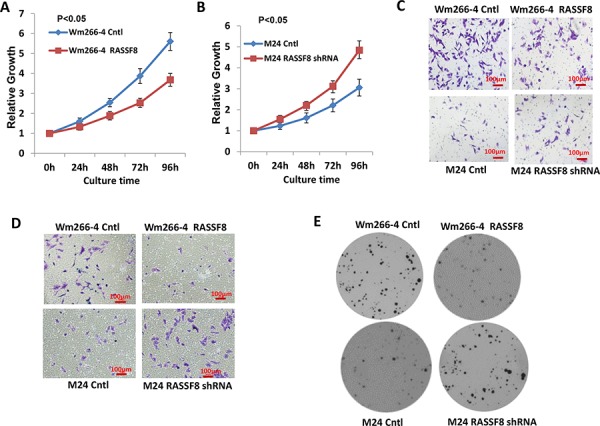
Function of RASSF8 in melanoma **A.** Overexpression of RASSF8 inhibited cell growth in Wm266-4 (*N* = 3). **B.** Knockdown of RASSF8 expression promoted cell growth in M24 (*N* = 3). **C.** Overexpression of RASSF8 inhibits cell migration in Wn266-4 whereas knockdown of RASSF8 increases cell migration in M24 (*N* = 3). **D.** Overexpression of RASSF8 inhibits cell invasion in Wn266-4 whereas knockdown of RASSF8 increases cell invasion in M24 (*N* = 3). **E.** Overexpression of RASSF8 inhibits colony formation of melanoma cells in soft agar (*N* = 3) whereas knockdown of RASSF8 increases colony formation of melanoma cells in soft agar (*N* = 3). RASSF8 plays inhibitory role in growth and mobility of melanoma cells.

To explore the functional role of RASSF8 in melanoma cells, Wm266-4, a melanoma cell line with low RASSF8 expression, was transfected with RASSF8 expression plasmid to overexpress RASSF8 and high RASSF8 expression cell clones, Wm266-4 RASSF8, were selected. We also developed knockdown models of RASSF8 in M24 cells, which normally have high RASSF8 expression, using RASSF8 shRNA and subsequently selected low RASSF8 expression cell clone M24-RASSF8 shRNA. Functional assays were also performed to compare colony formation in soft agar, cell growth, migration and invasion: Wm266-4 control, Wm266-4 RASSF8, M24 control, M24 RASSF8 shRNA, Wp-0614 Cntl, Wp-0614 RASSF8, M101 Cntl and M101 shRNA. Our results demonstrated significantly slower growth of Wm266-4 RASSF8 than Wm266-4 Cntl cells (Figure 2A), and higher growth of M24 RASSF8 shRNA versus M24 Cntl cells (Figure 2B). Similar results were observed in Wp-0614 Cntl and Wp-0614 RASSF8, M101 Cntl and M101 shRNA (Supplementary Figure 4A and 4B). In addition, we observed that RASSF8 expression is inversely correlated with cell migration and invasion (Figure 2B and 2C, Supplementary Figure 5 and 6). Results from the clonogenic assay show more colonies formed by M24 RASSF8 shRNA cells than M24 Cntl group, and significantly less colonies formed by Wm266-4-RASSF8 compared to Wm266-4 Cntl cells (Figure 2D). Similar results were observed in Wp-0614 Cntl vs Wp-0614 RASSF8, and M101 Cntl vs M101 shRNA treated (Supplementary Figure 7 and 8). These results suggest that RASSF8 expression has a tumor suppressor role in melanoma progression.

### Overexpression of RASSF8 induced cell apoptosis

**Figure 3:**
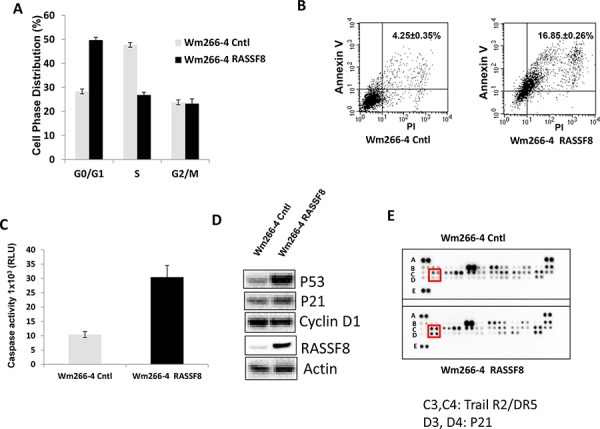
Overexpression of RASSF8 induced cell apoptosis by regulating P53-P21 pathway **A.** Cell cycle G1/S stage was arrested when RASSF8 was overexpressed. **B.** Apoptosis was induced when RASSF8 was overexpressed in melanoma cell. **C.** Caspase activity was increased when RASSF8 was overexpressed in melanoma cell. **D.** The expression of P53 and P21 were induced by RASSF8 whereas Cyclin D1 was reduced by RASSF8. **E.** Apoptosis array assay showed that P21 and TRAIL R2/DR5 were induced in cell apoptosis caused by RASSF8.

To further explore the role of RASSF8 in melanoma cells, we examined cell cycle and apoptosis. Overexpression of RASSF8 induced G1/S arrest and apoptosis in melanoma cells (Figure 3A and 3B). Caspase activity was significantly increased by overexpression of RASSF8 in Wm266-4 cells (Figure 3C), and was decreased by knockdown of RASSF8 expression in M24 cells (Supplementary Figure 9A). To further understand the underlying mechanism for apoptosis induced by RASSF8 expression, we performed western blot analysis to assess expression of P53, P21 and Cyclin D1 in Wm266-4 cells. As shown in Figure 3D while RASSF8 induced the expression of P53 and P21, it downregulated Cyclin D1 expression in Wm266-4 cells; these observations were confirmed by RASSF8 knockdown resulting in opposite signaling pathways; RASSF8 knockdown in M24 cells lead to reduced P53 and P21 expression, and increased Cyclin D1 expression (Supplementary Figure 9B). To further confirm whether P53-P21 apoptosis pathway was involved in RASSF8-induced apoptosis, we performed analysis using a human apoptosis array that indicated induction of P21 and TRAIL R2/DR5 in Wm266-4-RASSF8 cells (Figure 3E). In summary, these results support the findings of apoptosis induced through RASSF8 overexpression regulating the P53-P21 pathway.

### RASSF8 inhibits P65

**Figure 4:**
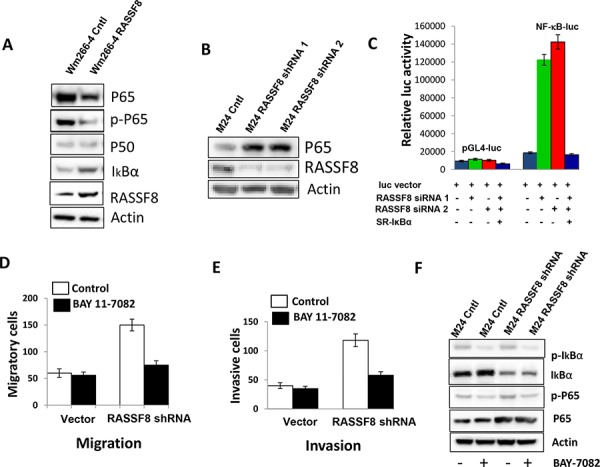
P65 was regulated by RASSF8 in melanoma cell line **A.** Overexpression of RASSF8 in Wm266-4 cells (Wm266-4-RASSF8) reduced expression of P65 and p-P65 but increased expression of IκBα. **B.** Knockdown of RASSF8 in M24 cells (M24-RASSF8 shRNA) increased P65 expression. **C.** NF-κB luciferase was activated by knockdown of RASSF8, and the activity was blocked by super-repressor IκBα in M24 cells. **D.** Pretreatment of NF-κB inhibitor BAY 11-7082 significantly blocked migration of Wm266-4 which has low RASSF8 expression. **E.** Pretreatment of NF-κB inhibitor BAY 11-7082 significantly blocked invasion of Wm266-4, which has low RASSF8 expression. **F.** p-IκBα and p-65 were more inhibited in M24 shRNA than that in M24 Cntl, whereas no change of IκBα was seen when cells were treated by BAY 11-7082. RASSF8 inhibited cell migration and invasion by regulating P65 expression.

Previous studies have demonstrated RASSF8 depletion leading to the loss of adheren junction (AJ) components, β-catenin and P65 from sites of cell–cell contact followed by their relocalization to the nucleus (16). Next, we performed western blot analysis to explore the relationship between RASSF8 and expression of P65, p-P65, P50 and IκBα in Wm266-4 Cntl and Wm266-4 RASSF8. Compared to Wm266-4 Cntl, expression of P65 and p-P65 was reduced while IκBα expression was increased in Wm266-4 RASSF8 (Figure 4A). There was no significant change of P50 expression in Wm266-4 Cntl and Wm266-4 RASSF8 (Figure 4A). Consistent results were observed in assessing Wp-0614 control versus Wp-0614 RASSF8 (Supplementary Figure 10A). Expression of P65 increased when RASSF8 was knocked down by RASSF8 shRNA in M24 and M101 cells (Figure 4B and Supplementary Figure 10B). IL-6 mRNA expression (downstream target of P65) in M24 RASSF8 shRNA treated was significantly increased compared to M24 Cntl (Supplementary Figure 11). Melanoma TCGA data analysis also showed that P65 mRNA expression in distant melanoma metastasis is significantly higher than that in lymph node regional melanoma metastasis (Supplementary Figure 2B). Luciferase assay indicated activation of NF-κB luc by RASSF8 siRNAs and its inhibition by super-repressor IκBα (SR-IκBα) [35] (Figure 4C). To investigate if RASSF8 exerted a functional role by regulating P65 expression, M24 and M24RASSF8 shRNA cells were pretreated by NF-κB inhibitor BAY 11-7082 [17, 18], and then cell migration and invasion were examined. As shown in Figure 4D and 4E, migration and invasion of M24-RASSF8 shRNA were significantly reduced by BAY 11-7082 compared to that of M24 control. p-IκBα and p65 were more inhibited in M24 shRNA than that in M24 Cntl, while no change in IκBα was observed upon treatment by BAY 11-7082 (Figure 4F). These findings indicate that RASSF8 exerts its function by regulating P65 in melanoma cells.

### RASSF8 methylation in melanoma

**Figure 5:**
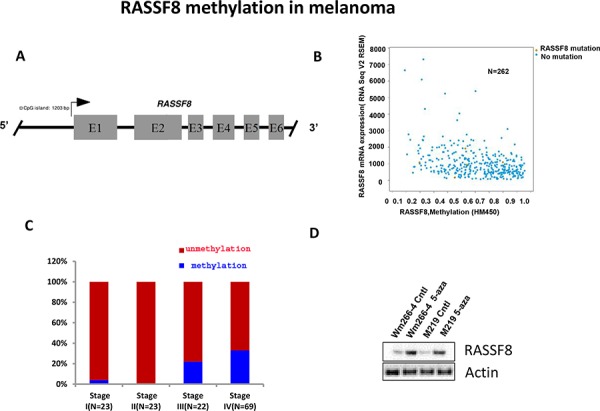
Methylation RASSF8 in melanoma **A.** The 1203bp CpG site in the promoter region of RASSF8. **B.** TCGA data showed an inverse relationship between RASSF8 methylation and RASSF8 mRNA expression in melanoma. **C.** RASSF8 methylation was higher in stage III/IV advanced melanomas than in stage I/II early melanomas. **D.** The expression of RASSF8 in metastatic melanoma lines with low RASSF8 expression was induced by treatment with 5-aza for 72 hrs.

Epigenetic regulation plays an important role in gene expression, especially through methylation of specific gene promoter regions [19, 20]. As illustrated in Figure 5A, there is a 1203bp CpG island in the promoter region of the RASSF8 gene. TCGA data analysis show significantly higher hypermethylation of RASSF8 in metastatic melanoma tissues, than earlier stage tumors (Figure 5B). To determine whether RASSF8 promoter methylation plays a role in progression of melanoma, MS-PCR was performed to assess specific CpG site methylation of the RASSF8 promoter using tissue specimens from different AJCC stages I-IV. Analysis of the MS-PCR results revealed higher RASSF8 methylation level in stages III and IV metastasis compared to primary tumors stage 1/II (Figure 5C). To further validate expression of RASSF8 is regulated by its CpG methylation, M219 and Wm266-4 were treated by 5-aza for 72 hrs and assessed by western blot. As shown in Figure 5D, expression of RASSF8 was induced by treatment of 5-aza. These results suggest that RASSF8 promoter region methylation regulates its expression in melanoma during tumor progression.

### RASSF8 protein expression in melanoma tissues and TMA

**Figure 6:**
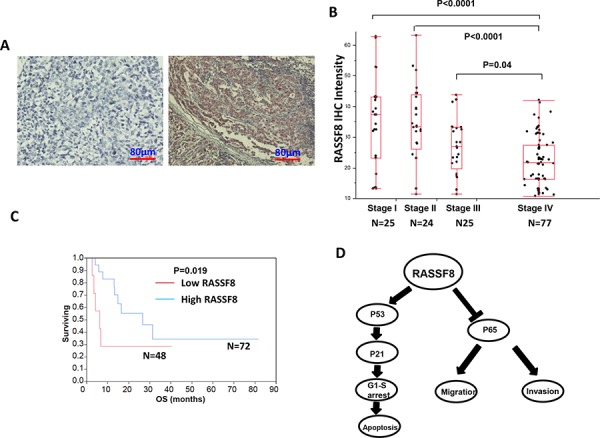
RASSF8 expression and clinical role in melanoma **A.** The representative photographs of negative control and positive expression of RASSF8 in melanoma tumors. **B.** RASSF8 expression was lower in stage III/IV advanced melanomas than in stage I/II early melanomas. **C.** Overall survival (OS) was better when RASSF8 expression was high. **D.** Schematic model for regulation of P53-P21 pathway and P65 expression by RASSF8.

To examine RASSF8 protein expression in melanoma tissues of different AJCC stages, we performed IHC in our well clinically annotated melanoma PEAT and TMA. Representative photographs are shown in Figure 6A. Results from IHC show a significantly lower RASSF8 expression in AJCC stage IV melanomas, compared to AJCC stage I, II and III melanomas (Figure 6B). Representative photographs of TMA IHC are included in Supplementary Figure 12A. RASSF8 expression appears significantly lower in stage IV than that in stage III melanomas in the TMA (Supplementary Figure 12B). As shown in the Kaplan-Meier survival analysis, patients with high RASSF8 expression (AJCC stage IV) have a higher survival rate than patients with low RASSF8 expression (Figure 6C). These findings support that RASSF8 plays a major role in the progression of melanoma metastasis

### Knockdown of RASSF8 enhanced growth of xenograft tumors in nude mice

**Figure 7:**
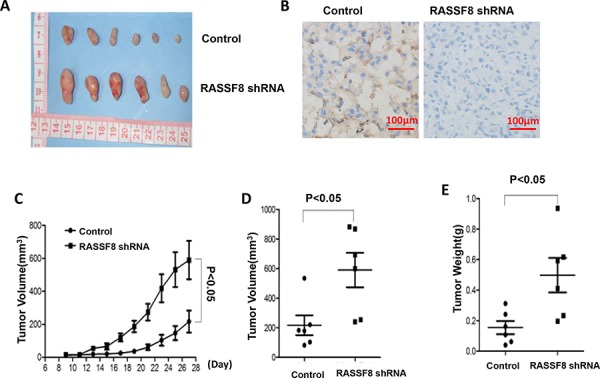
Growth of tumor xenografts in nude mice: comparison of M24 control and M24 RASSF8 shRNA **A.** Tumor xenografts. **B.** Representative photograph of RASSF8 IHC of xenograft tumor. **C.** Growth curve of xenograft tumors. **D.** Volume of xenograft tumor. **E.** Final weight of xenograft tumors. Xenograft tumors grew faster when RASSF8 expression was knocked down by RASSF8 shRNA.

To assess whether RASSF8 suppresses the tumorigenic properties of cell lines, M24 control and M24-RASSF8 shRNA were injected subdermally into the thigh of male nude-BALB/c mice (6 mice/cell line). Growth and size of tumor increased when RASSF8 was inhibited by RASSF8 shRNA. Inhibition of RASSF8 also increased the volume and weight of tumors (Figure 7). Here we have demonstrated RASSF8 knockdown leading to the significant increase in growth rate and tumorigenicity of a human melanoma cells in a nude mice xenograft model.

## DISCUSSION

Not only do N-terminal RASSF proteins (RASSF7-10) differ from the classical RASSF (RASSF1-RASSF6) members, but they also represent a newly defined set of potential Ras effectors. RASSF7-10 have been linked to key biological processes, including cell death, proliferation, microtubule stability, promoter methylation, vesicle trafficking, and response to hypoxia. In our previous study we showed repression of RASSF1A gene in a significant number of melanomas and suggested the potential role of CpG promoter region hypermethylation in the transcriptional inactivation of the RASSF1A gene in malignant melanoma [10]. In the present study, for the first time, we report RASSF8 tumor suppressor role by regulating P65 expression and P53-P21 pathway in melanoma; here we have demonstrated the correlation between the methylation and expression of RASSF8 gene, corresponding to aggressive tumor progression and function in melanoma. RASSF8 has previously been described as a candidate tumor suppressor gene in lung cancer [15, 21]. Lock et al. confirmed that RASSF8 inhibits cell growth and regulates the Wnt and NF-κB signaling pathways in lung cancer [16]. However, no functional studies had been conducted on RASSF8 in melanoma until now. Here, we have shown RASSF8-induced P65 expression, leading to inhibition of growth, migration and invasion of melanoma cells. RASSF8-induced P53 expression activates expression of P21, which is a potent cyclin-dependent kinase inhibitor (CKI). Subsequently, P21 binds to and inhibits the activity of cyclin-CDK2, -CDK1, and -CDK4/6 complexes, thus functioning as a regulator of cell cycle progression at G1 [22, 23]. Increase of P53 and P21 expression causes G1 arrest and cell apoptosis. Further manifesting these findings, our data supports the notion that overexpression of RASSF8 induces cell apoptosis via regulation of the P53-P21 pathway [22].

A previous study showed that RASSF8 was related to NF-κB pathway and may be required to maintain actin cytoskeletal organization [16]. Based on this study, P65 is lost from sites of cell–cell contact and gets relocated to the nucleus following RASSF8 depletion [16]. In our study, overexpression of RASSF8 inhibited growth, migration and invasion of melanoma cells by decreasing expression of P65 and regulating the P53-P21 pathway. Conversely, knockdown of RASSF8 shRNA was associated with a significant increase in P65 expression. The NF-κB Luciferase report assay showed that RASSF8 siRNA can activate the luciferase assay and the activity can be abolished by super-repressor IκBα. NF-κB inhibitor BAY 11-7082 significantly inhibited cell migration and invasion of M24 RASSF8 shRNA. Our results also showed expression of IL-6 (downstream target of P65) increasing upon RASSF8 reduced expression by RASSF8 shRNA in M24. These findings are consistent with the notion that RASSF8 regulates the expression of P65. RASSF8 exerts its effect on melanoma via regulation of P65 expression and the downstream target IL-6, thereby controlling tumor cell growth, migration and invasion.

Most members of RASSF family have CpG islands in the promoter region of their genes. In several previous studies, our team has demonstrated that RASSF1A promoter is more frequently methylated in metastatic melanomas compared to early stage melanomas [10, 11]. The expression of RASSF2A was completely silenced in gastric cancer cell lines as a result of promoter methylation and was restored by treating the cells with 5-aza-2-deoxycytidine [24]. Epigenetic inactivation of RASSF6 and RASSF10 is an extremely frequent event in the pathogenesis of childhood leukemia [25]. Our study is the first to demonstrate a direct correlation between RASSF8 methylation and progression, and an inverse correlation between RASSF8 expression and RASSF8 methylation. Re-expression of RASSF8 can be induced by treatment of 5-aza in melanoma cell lines. This is the first report on methylation of RASSF8 in cutaneous melanoma as a metastasis progression factor. Decreased expression of RASSF8 due to methylation was also found to be related to progression of melanoma. There is significantly lower RASSF8 expression in advanced melanoma compared to early stage melanoma. These studies support that RASSF8 acting as a tumor suppressor gene follows a similar pattern as the RASSF1 tumor suppressor gene.

Inhibition of RASSF8 increased the expression of P65 and decreased the expression of P53 and P21. Then, cell migration and cell invasion increased while cell apoptosis diminished. Xenograft studies confirmed that tumors grew faster in the RASSF8 knockdown group than in the control group. This study is a first report showing that inhibiting RASSF8 can increase the development of xenograft tumors *in vivo,* supporting its role as a tumor suppressor gene.

In conclusion, our results show that overexpression of RASSF8 can cause G1 arrest and induce apoptosis by regulating the P53-P21 pathway and that RASSF8 exerts its function via regulation of P65 expression (Figure 6D). Our study also confirms that methylation of RASSF8 promoter occurs in advanced melanoma. These results suggested that RASSF8 plays a tumor suppressor role in cutaneous melanomas. RASSF8 has the potential of being used as a theranostic factor to treat and predict the progression of cutaneous melanoma.

## MATERIALS AND METHODS

### Melanoma cell lines and treatment

We assessed expression of RASSF8 in 24 well-established, early-passaged cutaneous melanoma metastasis cell lines: M24, M101, FD0836, TG0873, M14, JH1173, ME7, M16, M20, CS0169, WP0614, ME35, BD0548, SR0488, AB0801, DP0574, M8133, LM0615, ME16, M21, ME9, M12, M211, and M13 established from AJCC stage III and IV melanoma patients who received surgery at JWCI.

Melanocyte line (HEM-M, Catalog #2216) was purchased from ScienCell (Carlsbad, CA), Wm266-4 from ATCC (Manassas, VA), and primary cell lines (WC00060, WC00080, WC00081) obtained from Coriell institute (Camden, NJ). Melanoma cell lines were maintained in appropriate culture medium supplemented with 10% fetal bovine serum (heat inactivated) and antibiotics at 37°C in a 5% CO2 atmosphere incubator, as previously described, and were used at early passages [26, 27]. Primary cells and melanocyte cell lines were cultured following manufacturer's instructions. For methylation assay studies, cultured cells were treated with 2 μmol/L 5-aza-2-deoxycytidine (Sigma-Aldrich, St.Louis, MO) dissolved in dimethyl sulfoxide (DMSO) (Sigma-Aldrich, St.Louis, MO) for 72 hrs with media changed every 24 hrs as previously described [10]. Control cells were incubated with DMSO under the same culture conditions.

### Tumor specimens

Approval for the use of human tissues was obtained from the joint IRB of John Wayne Cancer Institute and Providence Saint John's Health Center. Analysis was conducted on paraffin-embedded archival tissue (PEAT) specimens of cutaneous melanoma diagnosed at Providence Saint John's Health Center. Patients were staged using the current AJCC staging system for cutaneous melanoma (AJCC staging manual 7^th^ edition 2010).

### Tissue Microarrays

Tissue Microarrays (TMAs) were developed and were clinically well-annotated with >5yr follow-up, as previously described [28, 29]. AJCC stage III and IV melanoma TMAs included 268 distant organ metastases and 39 autologous stage III lymph node metastases, as well as 29 cancer free normal tissues from each respective organ as controls.

### Quantitative reverse transcription-PCR

Total extracted RNA (1 μg) was used for cDNA synthesis with Oligo(dT)20 primers (Thermo Fisher Scientific, Grand Island, NY). The cDNA was added to an optimized quantitative reverse transcription-PCR (qRT-PCR) mixture that contained 1X SYBR Green PCR master mix (Quanta Biosciences, Gaithersburg, MD) and 500 nmol/L gene-specific primers. Assays were performed in triplicate on a CFX thermocycler (Bio-Rad, Hercules, CA). Specific gene plasmids for controls and Beta-2-microglobulin (β2MG) were used in all PCR analyses [26]. The value of qRT-PCR was normalized with Beta-2-microglobulin. The primers of RASSF8 and IL-6 (Integrated DNA Technologies, Inc., Coralville, Iowa) are listed in Supplementary Table 1.

### DNA extraction and bisulfite modification

For DNA isolation, 10 μM sections of PEAT from AJCC stage I-IV melanoma were used. A reference H&E slide was prepared from each sample to confirm tumor location for microdissection. Tumor cells were captured and microdissected onto caps using the PixCell II Laser Capture Microdissection (LCM) System (Life Technologies, Grand Island, NY). On cap sodium bisulfite modification was performed as previously described [10] by incubating the cap in 0.2 mol/L sodium hydroxide (NaOH) at 37°C for 15 min and then in a 4.5 mol/L sodium bisulfite solution containing sodium metabisulfite, hydroquinone, and NaOH (pH 5) at 60°C for 8 hrs. After incubation, the cap was rinsed with distilled water and soaked in 0.3 mol/L NaOH for 15 min. The film was then removed from the cap and immersed in lysis buffer containing proteinase K (Sigma-Aldrich, St.Louis, MO) and Tween 20 (Sigma-Aldrich, St.Louis, MO) at 50°C for 8 hrs, followed by proteinase K denaturation at 95°C for 15 min [30, 31].

### Methylation-specific PCR (MSP)

The methylation status of RASSF8 in specimens was assessed by MSP using two sets of primers for each gene, designed to amplify methylated (M) or unmethylated (U) DNA sequences. Primer sequences are listed in Supplementary Table 2. Bisulfite-modified DNA was subjected to quantitative real-time PCR amplification. PCR reactions contained SYBR Green (Quanta Biosciences, Gaithersburg, MD) and PCR reaction was carried out in a Bio-Rad CFX thermocycler system (Bio-Rad, Hercules, CA). Each assay included a universal unmethylated control and universal methylated control. Unmodified lymphocyte DNA was also used as a negative control for methylated and unmethylated PCR reactions. Melting curve analysis was performed to verify PCR product by comparing each sample to control samples. Specimens that showed amplification for methylated DNA were considered methylated [32, 33].

### Cell cycle analysis and cell apoptosis

Cell cycle analysis was carried out using flow cytometry. The cultured cells were harvested and washed with cold PBS. Cells were fixed in 70% ethanol at 4°C overnight and treated with RNase A (10 mg/ml) (Invitrogen, Grand Island, NY). Fixed cells were stained with propidium iodide (PI) (Invitrogen, Grand Island, NY) followed by incubation for 30 min at room temperature in the dark. The PI fluorescence of individual nuclei was measured using BD FACSVerse^TM^ flow cytometer (BD Bioscience, San Jose, CA). Data were analyzed with BD FACSuite software (Version: 23-12943-01 Rev. 01) [34].

For cell apoptosis analysis, cells were harvested, washed with PBS, and incubated with Alexa Fluor 488-conjugated annexin V and propidium iodide (PI) (BD Bioscience, San Jose, CA) for 30 min at room temperature. After incubation, cells were washed by Phosphate Buffered Saline Tween (PBST) and flow cytometry was performed to analyze cell apoptosis. Data were analyzed with BD FACSuite software.

### Immunofluorescence staining

M24, M24 shRNA and Wm266-4 RASSF8 cells were cultured in 8-well chamber slides (Lab-Tek, Division miles Laboratories Inc. Naperville, IL) to about 60% confluency. Cells were fixed with 4% paraformaldehyde (Thermo Scientific, Rockford, IL) for 15 min and then permeablized with PBS containing 0.1% Triton X-100 (Sigma-Aldrich, St.Louis, MO) for another 15 min. Slides were blocked by 5% BSA (Sigma-Aldrich, St.Louis, MO) for 30 min and incubated with RASSF8 Ab (1:100, Cat. # ab56921, Abcam, Cambridge, MA) at room temperature for 1 hr. Slides underwent three 5-min washes with PBS. Then, cells were stained with the secondary antibody (Ab), Alexa Fluor 488 goat anti-mouse IgG, (1:500, Life Technologies, Thermo Fisher Scientific, Inc., Waltham, MA) for another 30 min. Again, slides underwent three 5-min washings with PBS. Coverslips were mounted onto glass slides using Vectashield mounting medium containing DAPI (Vector Laboratories, Inc., Burlingame, CA) [35–37]. Cells were observed under a high-resolution microscope. As a negative control, RASSF8 was replaced with normal mouse IgG.

### Western blotting

Protein concentrations were determined using the Pierce BCA Assay (Thermo Scientific, Rockford, IL). Western blot was performed as previously described [15]. Membranes were immunoblotted overnight with primary mouse monoclonal anti-RASSF8 Ab (1:1000, Abcam, Cambridge, MA, Cat.# ab56921), anti-P53 (BD Biosciences, Cat.# 554294) and anti-P21 (BD Biosciences, Cat.# 556431), mouse monoclonal anti-P65 Ab (Santa Cruz, Cat.# sc-8008) and rabbit polyclonal anti-Cyclin D1 Ab (1:1000, Santa Cruz, Cat.# sc-753), rabbit polyclonal anti-P50 Ab (1:1000, Cell Signaling, Danvers, MA, Cat.# 3035) and rabbit anti-IκBα Ab, anti-p-65 (1:1000, Cell Signaling, Danvers, MA, Cat.# 9936 and 3033).

After immunoblotting, the membranes were washed 3 times with PBST and incubated for 1 hr with horseradish peroxidase-conjugated goat anti-mouse Ab (1:5000, Santa Cruz) or horseradish peroxidase-conjugated rabbit anti-rabbit Ab (1:5000, Santa Cruz). Immunoreactive bands were visualized with the SuperSignal West Dura Extended Substrate Kit (Thermo Scientific, Rockford, IL) and the densities of protein bands were quantified by Alpha Ease FCTM software (Version 3.1.2, Alpha Innotech Corp, San Leandro, CA).

### Cell transfection

To investigate the functional role of RASSF8 in melanoma cells, we used a RASSF8 expression plasmid to transfect melanoma cell line Wm266-4, Wp-0614 (low RASSF8 expression) and subsequently selected stable cell clones with high RASSF8 expression (Wm266-4 RASSF8, Wp-0614 RASSF8). We then used RASSF8 small hairpin RNA (RASSF8 shRNA) (Sigma-Aldrich) to transfect melanoma cell line M24, M101 (high RASSF8 expression) and subsequently selected stable cell clones with low RASSF8 expression (M24-RASSF8 shRNA, M101 RASSF8 shRNA). We used these cell lines to assess the role of RASSF8 in functional studies, such as growth, migration, invasion and soft agar. The RASSF8 shRNA 1 sequence is CCGGCGGACTATGGAAAGTGGTCTT CTCGAGAAGACCACTT TCCATAGTCCGTTTTTTG. The RASSF8 shRNA 2 sequence is CCGGCCTGTTAGGTTACATCTGCTACTCGAGTAGC AGATGTAACCTAACAGGTTTTTTG.

To knockdown RASSF8 expression in M24 cells, M24 cells were seeded at 2.5–3.0 × 10^5^ cells/60 mm dishes and transfected with 100 nmol/L (final) of RASSF8 siRNA and negative control (GE Dharmacon, Lafayette, CO) using the JetprimeTM Transfection Reagent (VWR International, Radnor, PA) for 48 hrs. Target sequence of RASSF8 siRNA 1 is GAAGAGGAAATTGTCCGTCTA. Target sequence of RASSF8 siRNA 2 is CACCAAACGCTTACAGGACAA.

### Cell growth and soft agar colony formation

Cell proliferation and viability were assessed by the 3-(4,5-dimethylthiazol-2-yl)-2,5diphenyltetrazolium (MTT) Assay (Sigma-Aldrich). A soft agar colony formation assay was performed using six-well culture plates. The number of clones was counted after 18 days [34]. Each experiment was performed in triplicate [38].

### Cell migration and invasion assay

Briefly, 10^4^ cells were plated on the top of the Boyden chamber inserts (BD Biosciences, San Jose, CA). Serum (10%) was used as the chemoattractant. To rule out the effect of cell proliferation, 2 μg/ml mitomycin C (Sigma-Aldrich, St.Louis, MO) was added to the cells. Cells on the lower surface of the inserts were stained and counted using a light microscope. For invasion assays, inserts were coated with a thin layer of Matrigel basement membrane matrix [39]. The cells on the lower surface were stained with crystal blue and counted in 4 randomly selected fields.

### Immunohistochemistry (IHC)

IHC was performed on 5 μm PEAT specimens that had been incubated overnight at 37°C. RASSF8 protein expression was assessed using mouse monoclonal anti-RASSF8 Ab (Abcam, Cambridge, MA, Cat.# ab56921) at a dilution of 1:100. IHC was performed using an optimized protocol. Slides were deparaffinized, rehydrated and washed in 1X PBS. Antigen retrieval was performed with 1X citrate buffer (Sigma-Aldrich, St.Louis, MO) at 100°C for 10 min and then incubated in H2O2 (Sigma-Aldrich, St.Louis, MO) at room temperature to block endogenous peroxidase. Separate slides were incubated in primary Ab against RASSF8 overnight in a 4°C humid chamber followed by 1 hr incubation with secondary biotinylated link Ab. The reaction for RASSF8 was developed using a labeled streptavidin biotin (LSAB) method (LSAB+ Kit; Dako, Carpinteria, CA) and visualized using VIP Substrate Kit (Vector Laboratories, Burlingame, CA). The negative controls consisted of sections treated with mouse serum alone (Santa Cruz Biotechnology). The sections were counterstained with hematoxylin (Sigma-Aldrich). A photograph of each IHC-stained section was taken for analysis using a Nikon Eclipse Ti microscope and NIS elements software (Nikon, Melville, NY). Staining density was determined by Image J software (http://rsbweb.nih.gov/ij/). After adjustment for background on each selected field, the density of the individual melanoma specimen was quantified and given a numerical value from 0–255. Melanoma specimens were tested in duplicate, and the average of the two staining intensity values was used for statistical analysis.

### Melanoma TCGA assay

Datasets in melanoma were downloaded from the Cancer Genome Atlas (TCGA) data portal (http://tcgadata.nci.nih.gov) in May, 2014. We extracted gene expression data using the “data matrix” tool provided by TCGA data portal.

### Nude mice for xenograft assays

24 nude mice (BALB/c background) (Experimental Animal Center, Chinese Academy of Sciences, Shanghai, China) were randomly divided into four groups and housed in air conditioned, light controlled animal facilities. Animal care and all experiments were in accordance with the institutional guidelines and were approved by the Animal Care and Use Committee of Huashan Medical Center, Fudan University in accordance with regulations of Institutional Animal Care and Use Committee. 1 × 10^6^ cells were inoculated subdermally into the right thigh. To test the tumorigenic properties of cells, M24 control and M24-RASSF8 shRNA were assessed (6 mice for each cell line).

Mice were weighed and subcutaneous tumors were measured after a week; tumor volume was obtained by the ellipsoid volume calculation formula: 0.5 × (length × width^2^).

### Statistical analysis

The results are given as mean ± SD. Student's *t*-test was used to calculate differences between the various study groups. The biological difference was considered statistically significant at *p* < 0.05.

## SUPPLEMENTARY FIGURES AND TABLES


