# Supplementary material for: RASSF8 regulates progression of cutaneous melanoma through nuclear factor-κb
Source: Oncotarget. 2015 Aug 17;6(30):30165–77. doi: 10.18632/oncotarget.5030 (PMC4745788; doi:10.18632/oncotarget.5030)
Supplement: Supplementary file 1 [file oncotarget-06-30165-s001.pdf]

SUPPLEMENTARY FIGURES AND TABLES

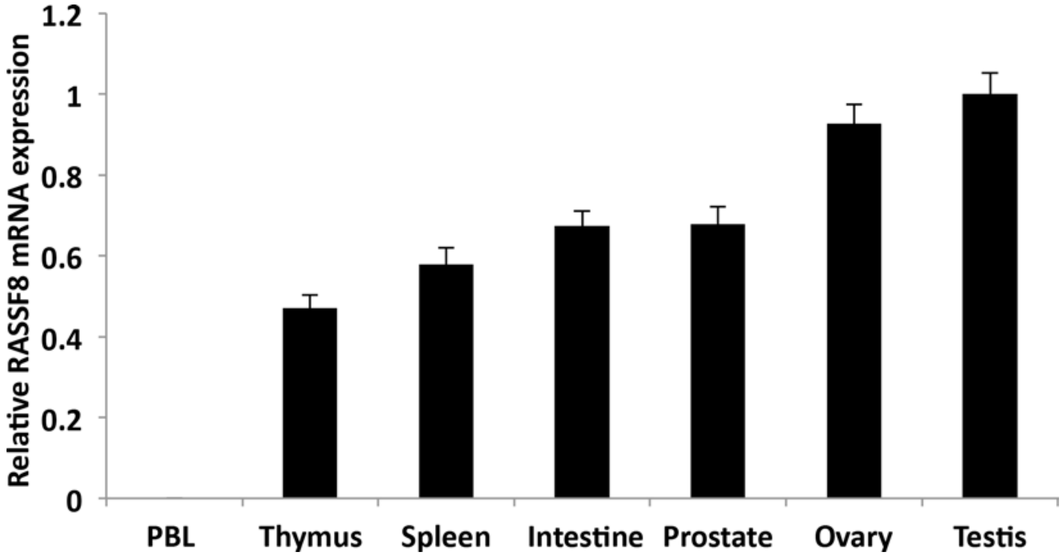

Supplementary Figure S1: RASSF8 expression in different organs. RASSF8 expression was observed in different human normal organs. There is higher RASSF8 mRNA expression level in ovary and testis compared to other organs.

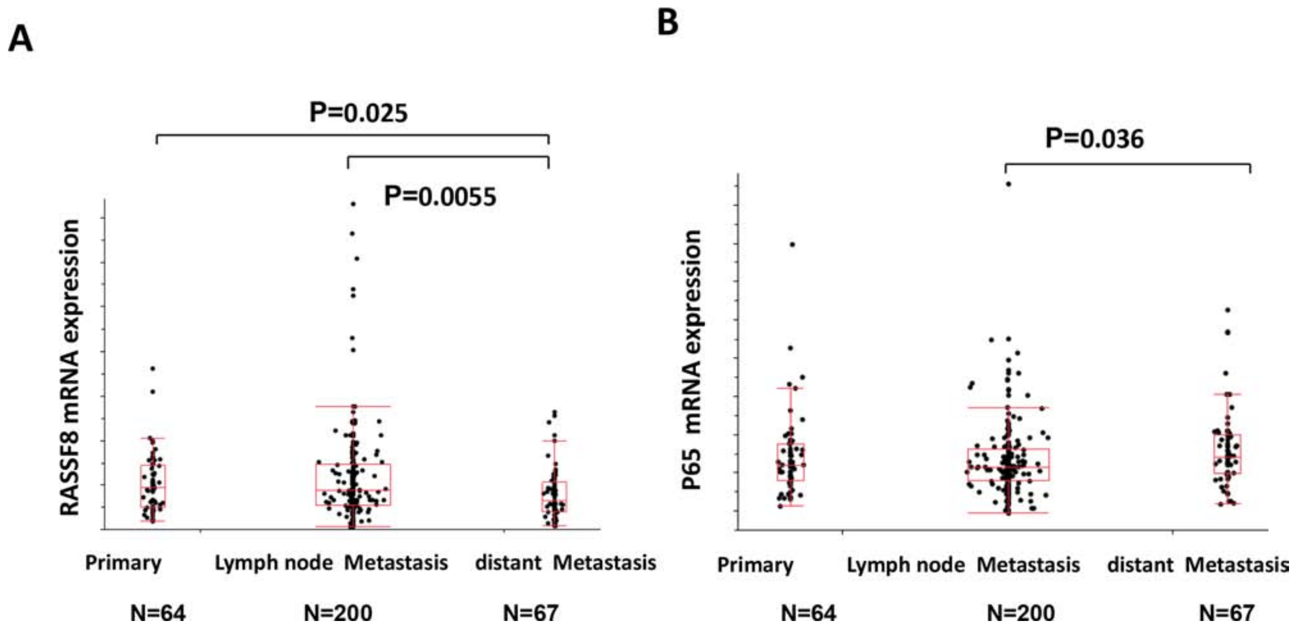

Supplementary Figure S2: RASSF8 mRNA expression and P65 mRNA expression in TCGA melanomas. A. RASSF8 mRNA expression in primary, lymph node metastasis and distant metastasis melanomas. There was significantly lower RASSF8 expression in distant metastasis melanomas than in primary melanoma and lymph node metastasis. **B.** P65 mRNA expression in primary, lymph node metastasis and distant metastasis melanoma. There is significantly higher P65 expression in distant metastasis melanoma than in lymph node metastasis.

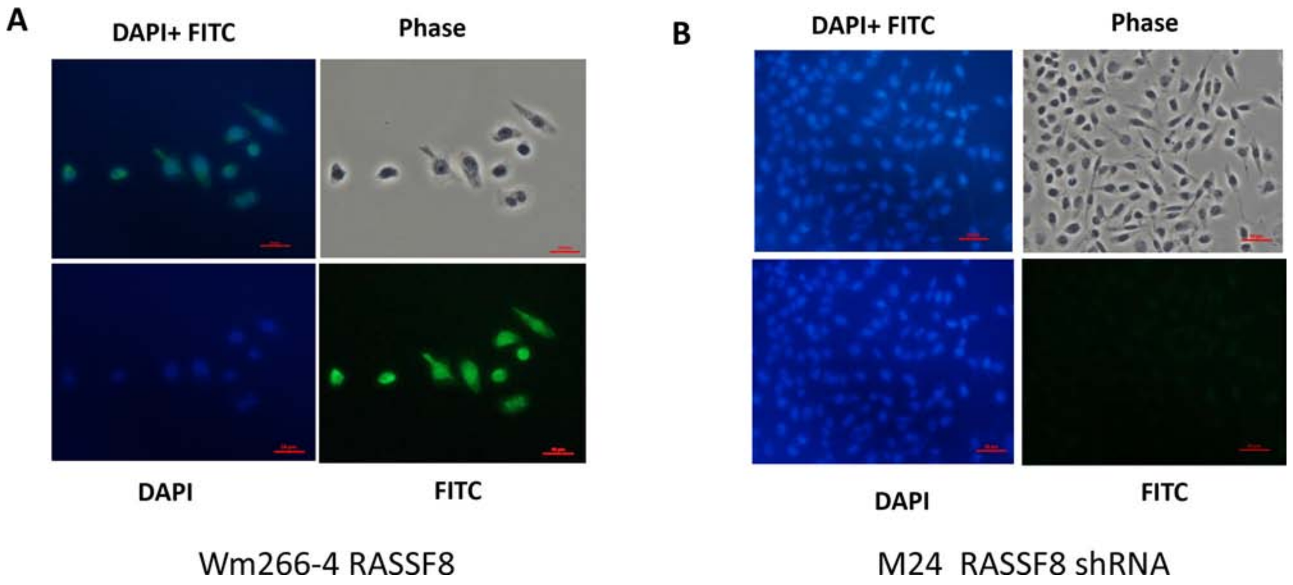

Supplementary Figure S3: Immunofluorescence staining of RASSF8 by mouse monoclonal anti-RASSF8 Ab (Abcam, Cambridge, MA, Cat.# ab56921) in RASSF8 overexpressed cells and knockdown cells. A. There was a strong fluorescence in RASSF8 overexpression cells. **B.** There was very weak fluorescence activity in RASSF8 knockdown cells.

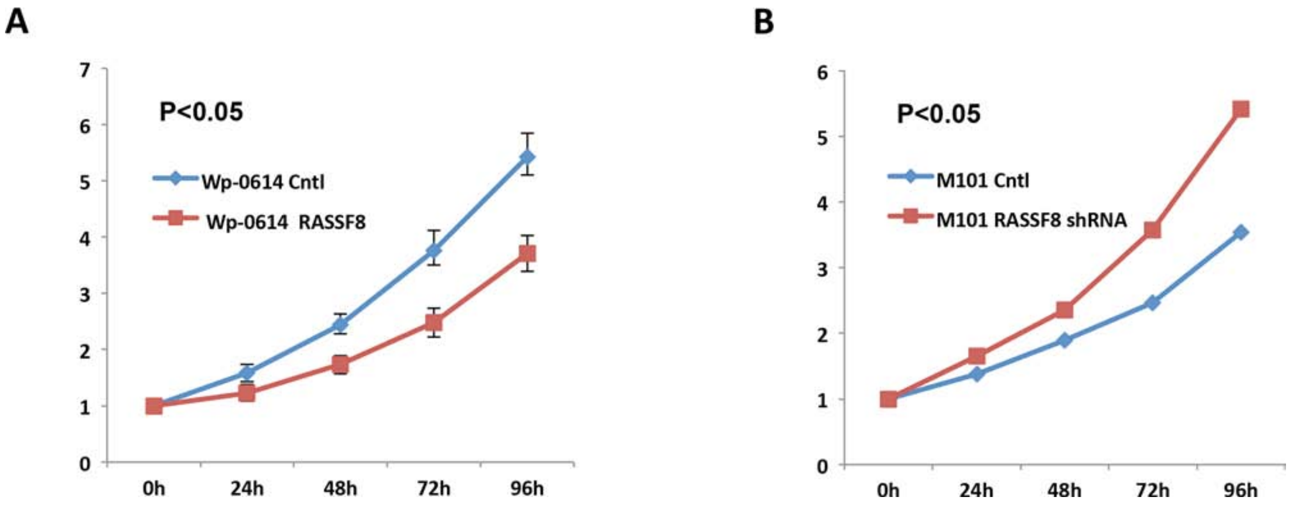

Supplementary Figure S4: Cell growth of Wp-0614 Cntl and Wp-0614 RASSF8, M101 Cntl and M101 shRNA. A. Overexpression of RASSF8 inhibited cell growth. **B.** Knockdown of RASSF8 expression promoted cell growth.

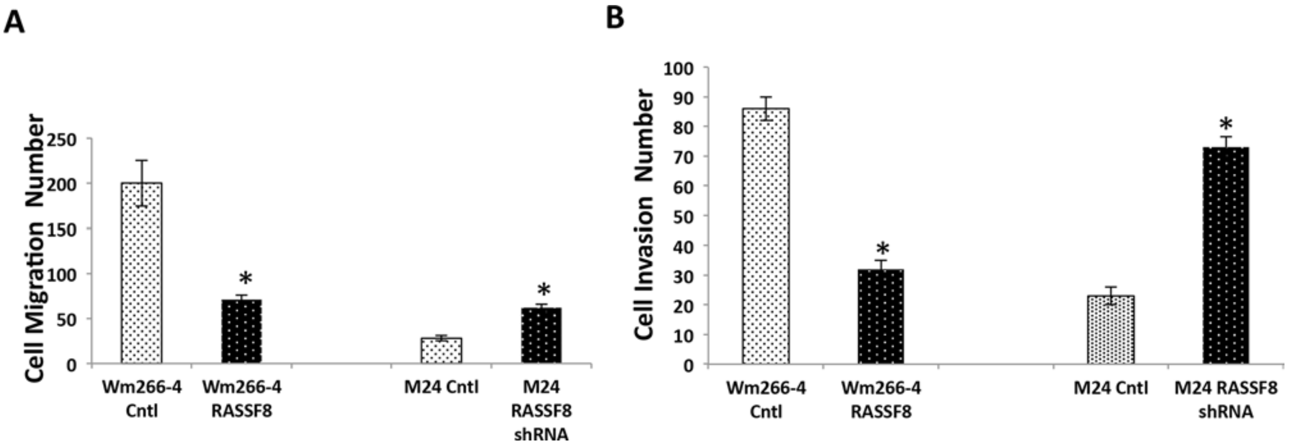

Supplementary Figure S5: Quantification of cell migration and invasion. **A.** Cell migration of Wm266–4 RASSF8 was significantly decreased compared to Wm266–4 Cntl respectively ($N = 3$), whereas cell migration of M24 shRNA was significantly increased compared to M24 Cntl respectively ($N = 3$) (*$p < 0.05$). **B.** Cell invasion of Wm266–4 RASSF8 was significantly decreased compared to Wm266–4 Cntl, respectively ($N = 3$), whereas cell invasion of M24 shRNA was significantly increased compared to M24 Cntl, respectively ($N = 3$) (*$p < 0.05$).

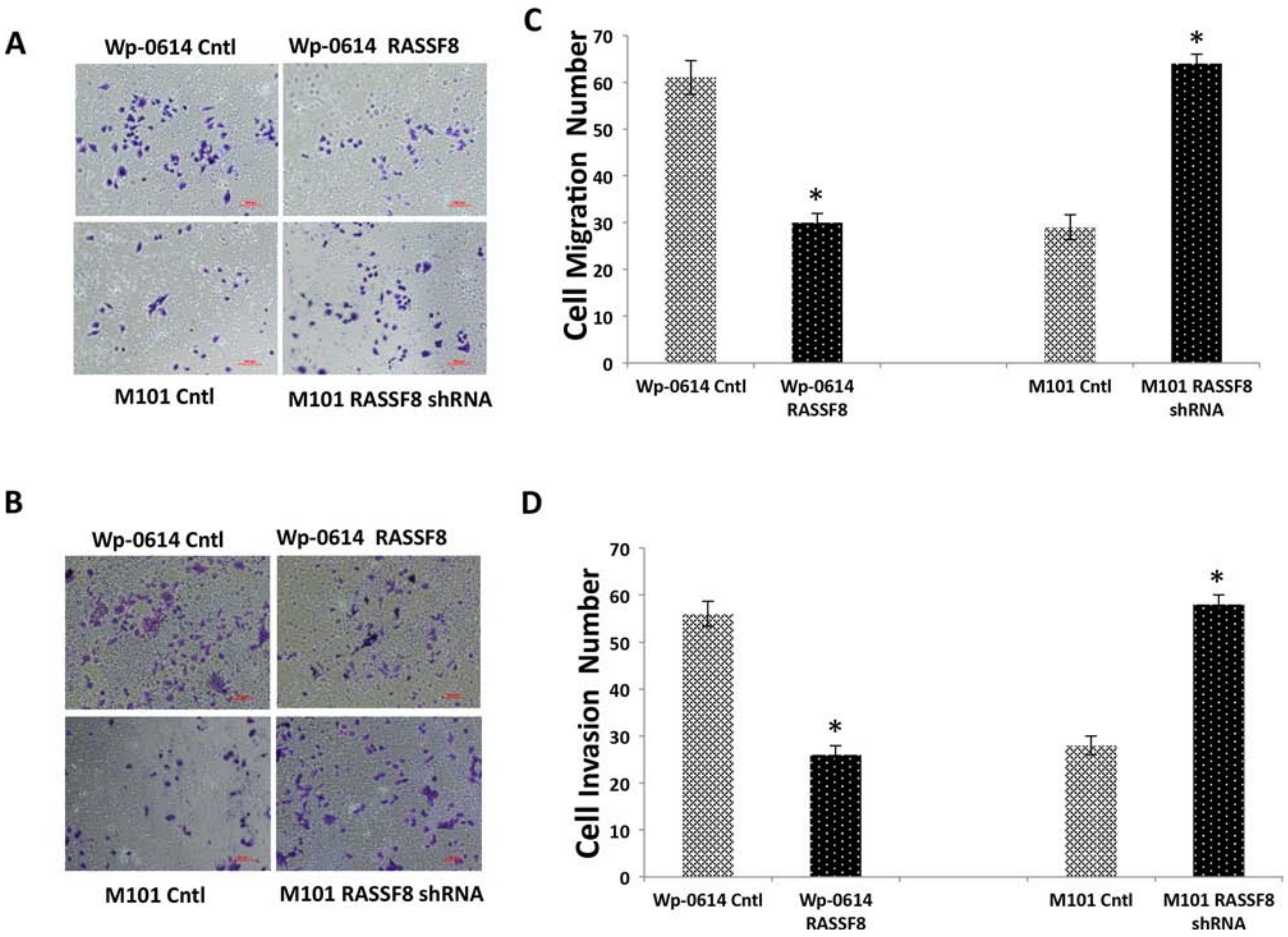

Supplementary Figure S6: Migration and invasion of Wp-0614 Cntl and Wp-0614 RASSF8, M101 Cntl and M101 shRNA. A. Migration analysis of Wp-0614 Cntl vs Wp-0614 RASSF8, M101 Cntl vs M101 shRNA. **B.** Invasion analysis of Wp-0614 Cntl vs Wp-0614 RASSF8, M101 Cntl vs M101 shRNA. **C.** Quantification of migration ($N = 3$). cell migration of Wp-0614 RASSF8 was decreased significantly compared to Wp-0614 Cntl whereas cell migration of M101 shRNA was increased significantly compared to M101 Cntl (*$p < 0.05$). **D.** Quantification of invasion ($N = 3$). Cell invasion of Wp-0614 RASSF8 was decreased significantly compared to Wp-0614 Cntl, whereas cell invasion of M101 shRNA was increased significantly compared to M101 Cntl (*$p < 0.05$).

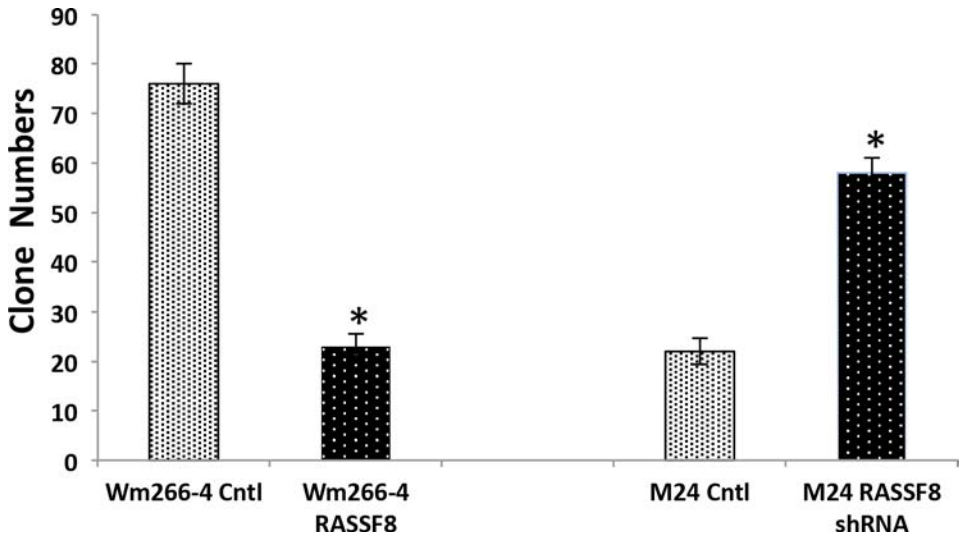

Supplementary Figure S7: Quantification of clones. Number of clones in Wm266–4 RASSF8 was significantly decreased compared to Wm266–4 Cntl ($N = 3$), whereas number of clones in M24 RASSF8 shRNA was significantly increased compared to M24 Cntl ($N = 3$,*$p < 0.05$).

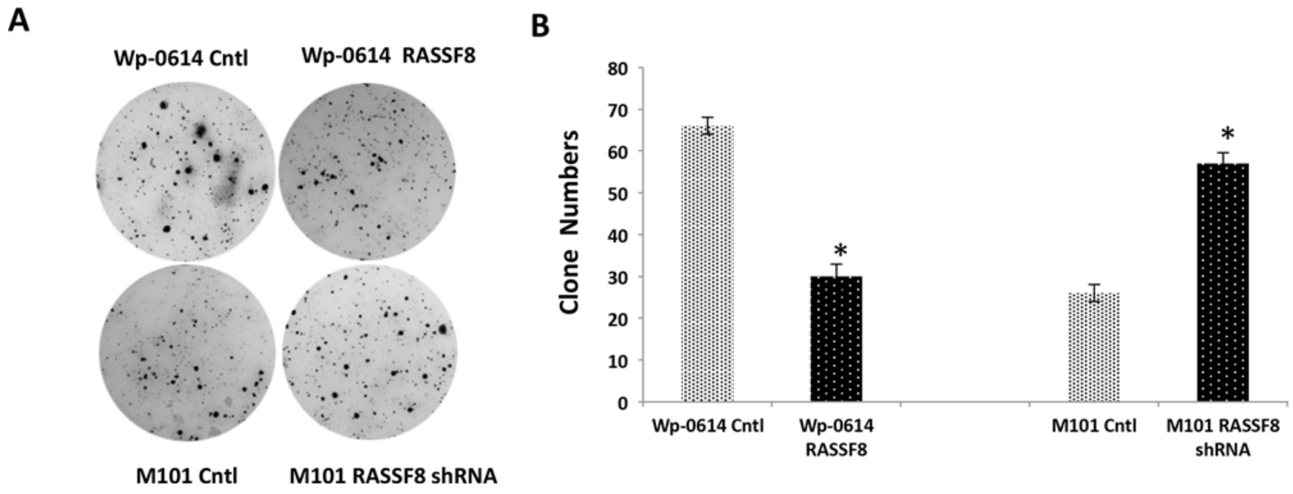

Supplementary Figure S8: Colony formation assay of Wp-0614 Cntl and Wp-0614 RASSF8, M101 Cntl and M101 shRNA. **A.** Colony formation of Wp-0614 Cntl vs Wp-0614 RASSF8, M101 Cntl vs M101 shRNA. **B.** Quantification of clones in Wp-0614 Cntl vs Wp-0614 RASSF8, M101 Cntl vs M101 shRNA. Number of clones in Wp-0614 RASSF8 was significantly decreased compared to Wp-0614Cntl ($N = 3$), whereas number of clones in M101 RASSF8 shRNA was significantly increased compared to M101 Cntl ($N = 3$, *$p < 0.05$).

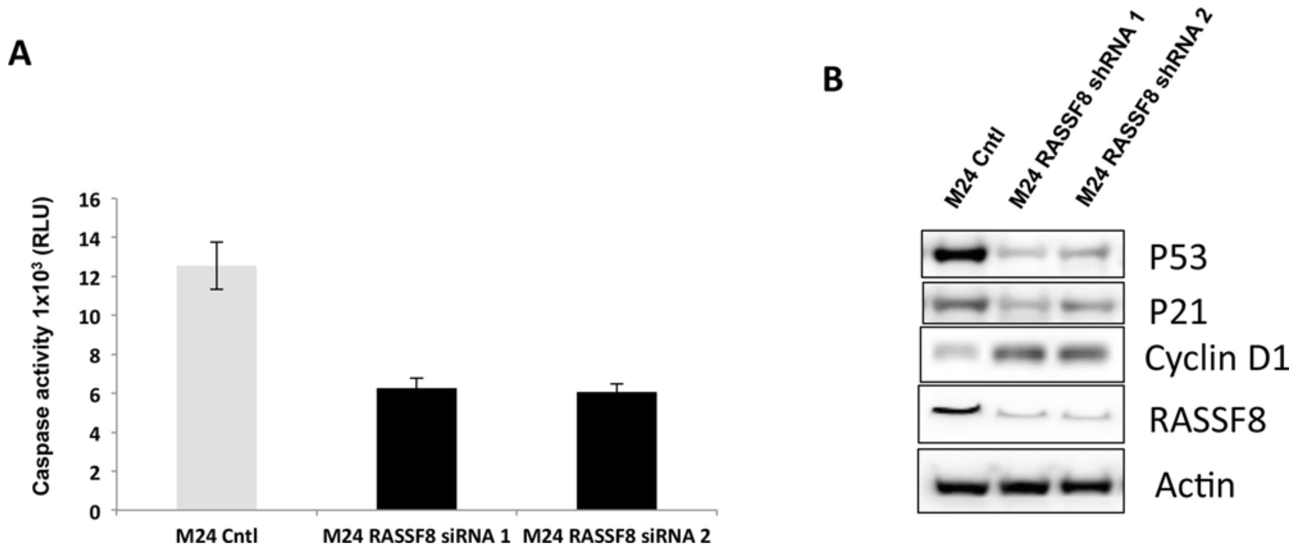

Supplementary Figure S9: RASSF8 regulated P53-P21 pathway. A. Caspase activity was reduced when RASSF8 was knocked down by RASSF8 siRNA in M24 cells. **B.** Expression of P53 and P21 was reduced while expression of Cyclin D1 was increased when RASSF8 was knocked down by RASSF8 siRNA in M24 cells.

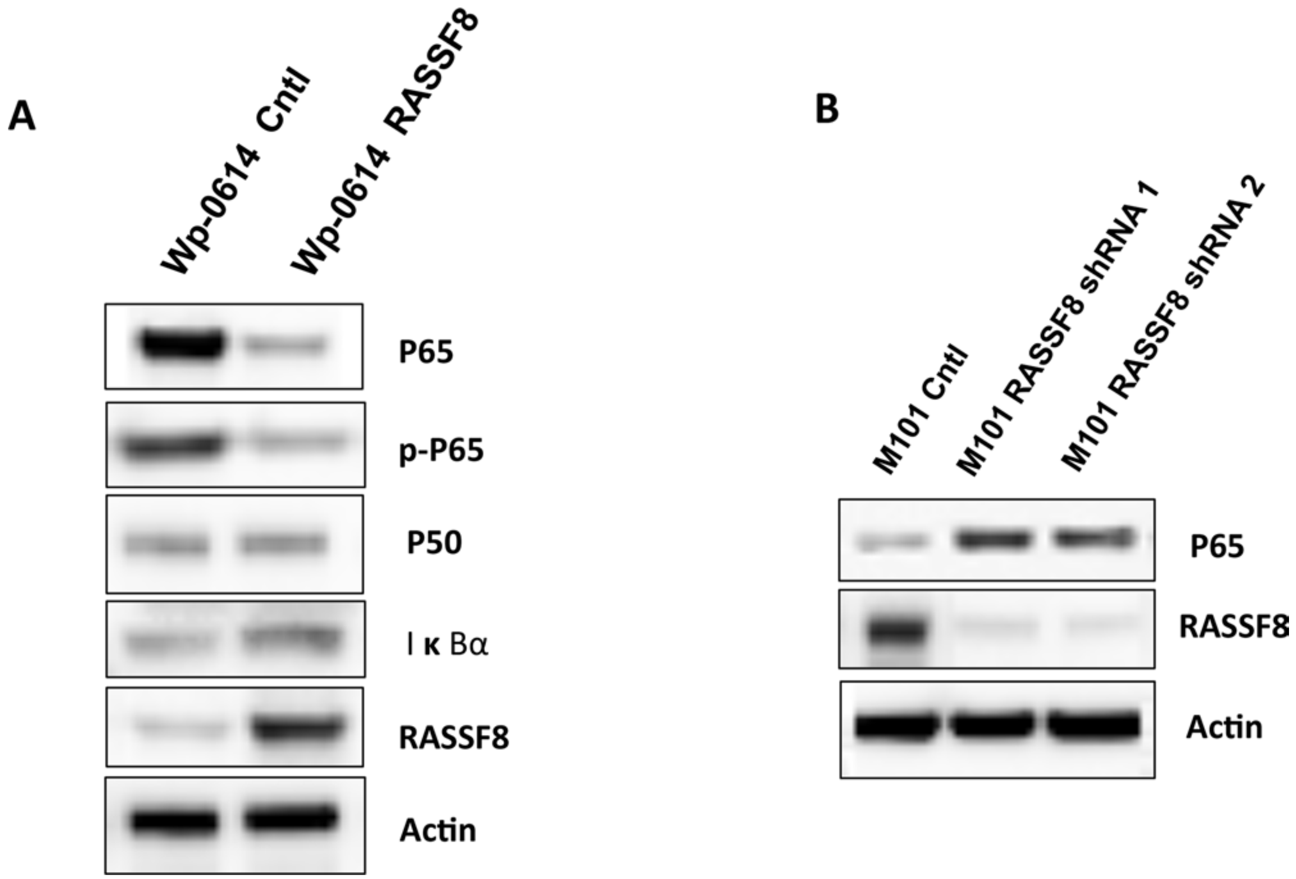

Supplementary Figure S10: RASSF8 regulated expression of P65, p-P65 and IκBα in melanoma cell lines. A. Overexpression of RASSF8 in Wp-0614 cells (Wm266–4-RASSF8) reduced expression of P65 and p-P65 but increased expression of IκBα. **B.** Knockdown of RASSF8 in M101 cells (M101-RASSF8 shRNA) increased P65 expression.

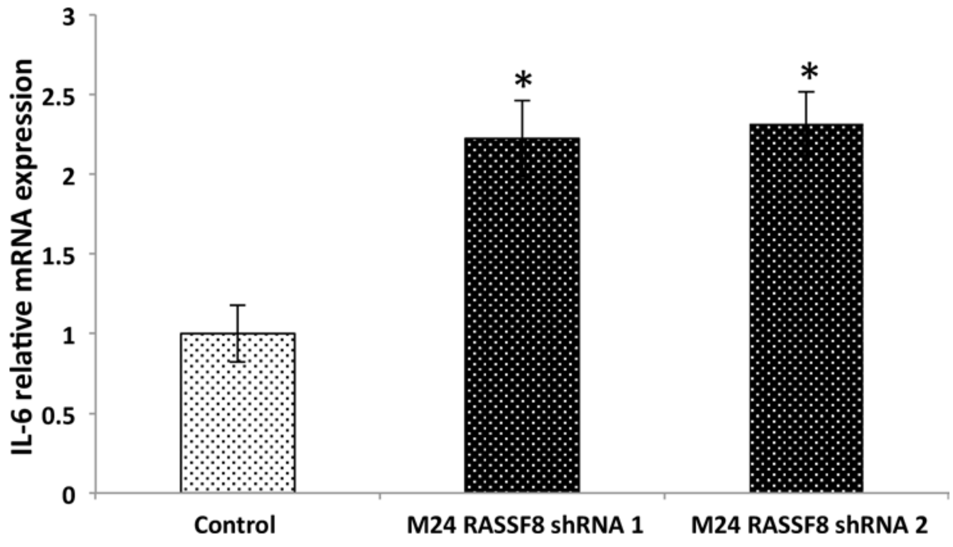

Supplementary Figure S11: IL-6 mRNA expression in tumors from control cells and RASSF8 shRNA cells. IL-6 mRNA expression was significantly increased in RASSF8 shRNA cells compared to control cells ($N = 3$, $^*p < 0.05$).

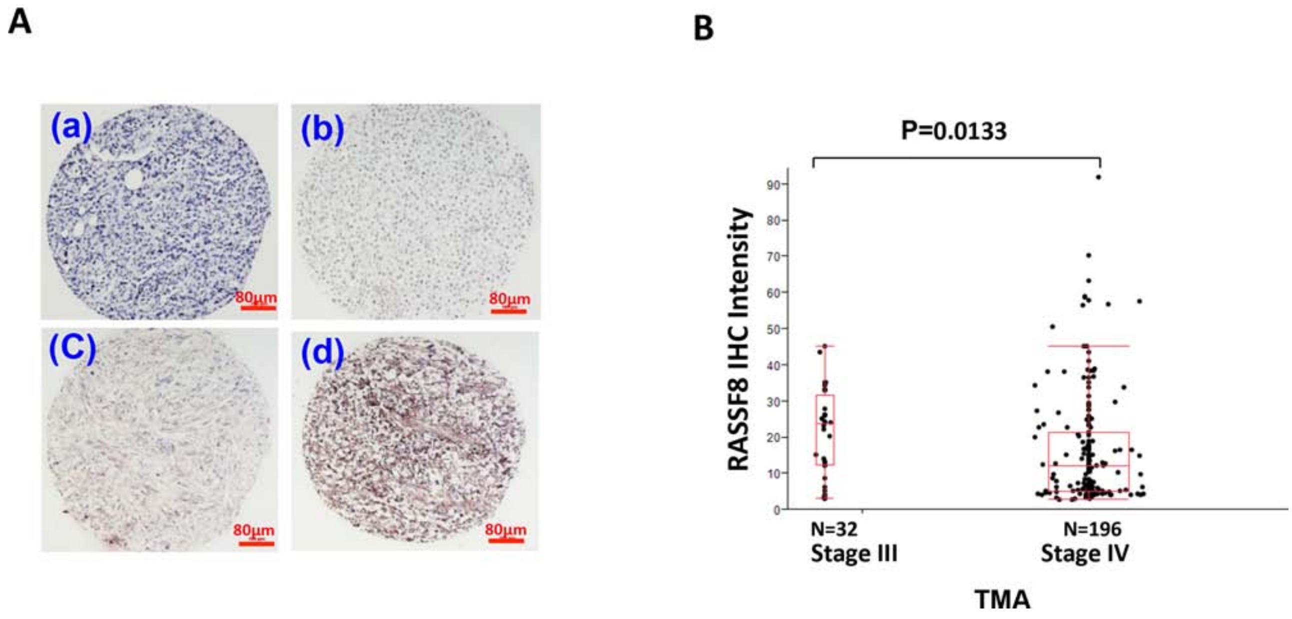

Supplementary Figure S12: RASSF8 Immunohistochemistry of TMA. A. Representative photographs of TMA. Negative (a); Low (b); Medium (c); High; and (d) RASSF8 expression. **B.** RASSF8 expression was significantly lower in stage IV than stage III melanomas.

Supplementary Table S1: Primers for RT-qPCR Assays

Biomarker	Forward	Reverse
RASSF8	CAAAGGGGAGATTGACA	TTCCTGTTCTTTGTCCTG
Beta-2-microglobulin	TGTCACAGCCCAAGATAG	CCAGCAAGCAGAATTTGGAA
IL-6	TCCAGAACAGATTTGAGAGTAGTG	GCATTTGTGGTTGGGTCAGG

Supplementary Table S2: RASSF8 Primers for Methylation Assays

Type	Forward	Reverse
M-1	CGTTTTTATTTTGTTTCGTCGC	CAATCCGCGACTTATATACCG
M-2	TTTCGTCGCGGGTTTTCG	CAATCCGCGACTTATATACCG
U	TTTGTTTTGTTTTTATTTTGTTTTG	CTTTTCAATCCACAACTTATATACCAC
